# Disabling symptoms associated with increased axillary temperature in patients with functional hyperthermia

**DOI:** 10.1186/s13030-024-00306-8

**Published:** 2024-03-26

**Authors:** Takakazu Oka

**Affiliations:** Department of Psychosomatic Medicine, International University of Health and Welfare Narita Hospital, 852 Hatakeda, Narita, Chiba 286-8520 Japan

**Keywords:** Functional hyperthermia, Fatigue, Fever-associated symptom, Psychogenic fever

## Abstract

**Background:**

I previously reported a case of functional hyperthermia (FH) in a patient with an axillary temperature just slightly above 37.0 °C who persistently requested treatment. Because the severity of her fatigue increased remarkably when her axillary temperature increased above 37.0 °C, she felt that the temperature of 37.0 °C was disabling. In the present study, I analyzed a larger number of patients with FH to investigate the incidence of disabling symptoms with increasing body temperature, the kinds of symptoms associated with increased body temperature, and the temperatures at which these symptoms became disabling.

**Main body:**

Twenty patients with FH (7 men, 13 women; mean age ± standard deviation, 31.2 ± 10.9 years) who visited my department were asked whether they had any disabling symptoms associated with an increase in axillary temperature and, if so, at what temperature the symptoms became disabling. Sixteen of 20 patients (80.0%) responded that they had such symptoms, which included worsening of general fatigue (*n* = 12, 75.0%), feelings that their brain did not work properly (*n* = 5, 31.3%), inability to move (*n* = 4, 25.0%), hot flashes/feeling of heat (*n* = 3, 18.8%), headache (*n* = 2, 12.5%), dizziness (*n* = 2, 12.5%) and anorexia (*n* = 1, 6.3%). The axillary temperatures at which patients felt worsening fatigue ranged from 37.0 °C to 37.4 °C in 7 of the 12 patients (58.3%) who experienced worsening fatigue. The patients also reported that the disabling symptoms, with the exception of headache, were not alleviated by antipyretics.

**Conclusions:**

Many patients with FH reported worsening fatigue as a disabling symptom associated with increased axillary temperature; more than half of those patients experienced worsening fatigue in the temperature range of 37.0 °C to 37.4 °C. These findings suggest that the reasons patients with FH consider 37 °C disabling and seek medical treatment are that physical symptoms such as fatigue worsen at 37 °C, although this temperature is assumed by many physicians to be within the normal range or just above the normal range of axillary temperature, and that most hyperthermia-associated symptoms are not alleviated by antipyretic drugs.

## Background

I previously reported a case of functional hyperthermia (FH) in a patient who complained of marked worsening of general fatigue when her axillary temperature exceeded 37.0 °C [1, 2]. She persistently asked for treatment of what she called “fever,” despite having an axillary temperature of 37 °C, which is generally considered to be normal. Through her treatment, I came to understand that her increased temperature-associated worsening of fatigue was so disabling that it was the reason she persistently sought treatment. When her fatigue decreased with pharmacotherapy, she did not appear to be bothered by her temperature, even though it exceeded 37.0 °C. If patients with FH have fever/hyperthermia-associated symptoms similar to hers, it is reasonable for them to request treatment, even though their low-grade fever/hyperthermia is not caused by any serious inflammatory disease and their temperature is just above the normal range of body temperature.

To confirm and extend my findings, the present study was conducted with a larger number of cases to investigate how many patients with FH develope disabling symptoms associated with an increase in axillary temperature, what kinds of symptoms were associated with an increase, and the temperature at which these symptoms became disabling.

## Methods

Twenty patients with FH (7 men, 13 women; mean age ± standard deviation [SD], 31.2 ± 10.9 years) who visited the Department of Psychosomatic Medicine at the International University of Health and Welfare Narita Hospital were asked whether they had any symptoms that appeared or worsened when their axillary temperatures increased and, if so, at what temperature (℃) disabling symptoms developed. They were also asked about the effectiveness of antipyretic drugs (non-steroidal anti-inflammatory drugs [NSAIDs]).

Most patients had undergone thorough medical evaluations at multiple medical facilities (range, 1 – 14 clinics and hospitals; mode, 5), had measured their axillary temperature multiple times with their own thermometer or the hospital's electronic thermometer, and had taken NSAIDs. In some hospitals, they had been asked to record their axillary temperature regularly. In my department, they were asked to record their axillary temperature four times a day for several days: in the morning, at noon, in the afternoon (around 4 p.m.), and just before going to bed.

In this study, FH was diagnosed based on the following criteria: (1) Maximal axillary temperature of 37.5℃ or more, given that measurement of axillary (versus oral) temperature is common in Japan and that the Infectious Diseases Control Law of Japan states “fever is defined as a body temperature of 37.5 °C or higher,” (2) no abnormal physical signs accounting for high temperature, (3) no abnormal urinary and blood test findings, e.g., urinalysis, complete blood count, C-reactive protein, erythrocyte sediment rate, anti-nuclear antibody, thyroid hormone, catecholamines, and cortisol, (4) no abnormal imaging study findings, e.g. chest X-ray, abdominal ultrasonography, magnetic resonance imaging, and gallium scintigraphy if necessary, and (5) failure of NSAIDs to attenuate high axillary temperature.

## Results

Patient demographics and clinical characteristics are shown in Table 1. The maximum temperature ranged from 37.5 °C to 39.0 °C (mean ± SD, 38.0 ± 0.4 °C). Eight patients had a continuous low-grade fever in the 37 °C range, and another 8 patients had a continuous low-grade fever and sometimes experienced an episodic high fever of 38 °C or higher.

**Table 1:** Patient demographics and clinical characteristics

Age, mean ± SD, years	31.2 ± 10.9
Sex, male:female, *n* (%)	7 (35):13 (65)
Maximal axillary temperature, ℃	
Mean ± SD	38.0 ± 0.4
Range	37.5 – 39.0
Fever duration	
Mode	2 years
Range	2 months – 7 years
Fever subtype, *n*	
1) Usually a normal temperature, but suddenly develops a high fever of 38 °C or higher	0
2) A low-grade fever in the 37 °C range persists for more than several weeks	8
3) A low-grade fever in the 37 °C range intermittently	4
4) A continuous low-grade fever in the 37 °C range and sometimes a high fever of 38 °C or higher	8
Visited psychiatrists, *n* (%)	13 (65%)
Final diagnosis of comorbid psychiatric disorders (includes duplicate diagnoses), *n*	
None except for functional hyperthermia	4
Depressive disorders	4
Adjustment disorders	3
Anxiety disorders	3
Somatic symptom and related disorders	2
Neurodevelopmental disorders	1
Post-traumatic stress disorder	1
Presence of physical disease, *n* (%)	8 (40%)
Comorbid physical diseases (includes duplicate diagnoses), *n*	
Premenstrual syndrome	5
Irritable bowel syndrome	3
Orthostatic dysregulation	2
Tension-type headache	2
Functional dyspepsia	1
Sleep apnea syndrome	1
Overactive bladder	1
Hypertension	1
Background factors, *n*	
Stressful events within the past six months before the onset of symptoms	18
Inflammatory events, such as viral infection, just before symptom onset	8
Both stressful events and inflammatory events before the onset of symptoms	7
Adverse childhood experiences	5

Background factors were determined from the patient's pre-examination form and medical interview at the first visit. Eighteen patients reported that they had high stress within the past 6 months before the onset of symptoms and that it might be associated with their low-grade fever. Two patients denied the involvement of psychosocial stress. One female patient was a doctor who developed symptoms while busy with her job, raising 2 young children, and doing housework. However, she did not find it stressful. One male patient who had a psychologically traumatic experience as a child was worried about interpersonal relationships at work and in his family, but he did not feel that this was stressful. In other words, these 2 patients exhibited characteristics of alexisomia, or difficulty in the awareness and expression of physical conditions [3]. Therefore, in the author’s opinion, all patients with FH in this study could be diagnosed with psychosocial stress-related psychogenic fever

Among the 20 patients, 16 (80.0%) responded that they had symptoms that developed with increased axillary temperature. These symptoms included worsening of general fatigue (*n* = 12, 75.0%), feelings that their brain did not work properly (*n* = 5, 31.3%), difficulty moving (*n* = 4, 25.0%), hot flashes/feeling of heat (*n* = 3, 18.8%), headache (*n* = 2, 12.5%), dizziness (*n* = 2, 12.5%), and anorexia (*n* = 1, 6.3%) (Table 2). Most patients responded that NSAIDs did not alleviate these symptoms or their high axillary temperature, what they called fever. Two patients, however, reported that NSAIDs improved their headache, despite failing to attenuate their fever.

**Table 2:** Disabling symptoms associated with increased axillary temperature

Symptom, *n* (%)	Patients *N* = 16
Worsening of general fatigue	12 (75.0)
Feelings that the brain does not function properly	5 (31.3)
Difficulty moving	4 (25.0)
Hot flash/feeling of heat	3 (18.8)
Headache	2 (12.5)
Dizziness	2 (12.5)
Anorexia	1 (6.3)

**Fig. 1:**
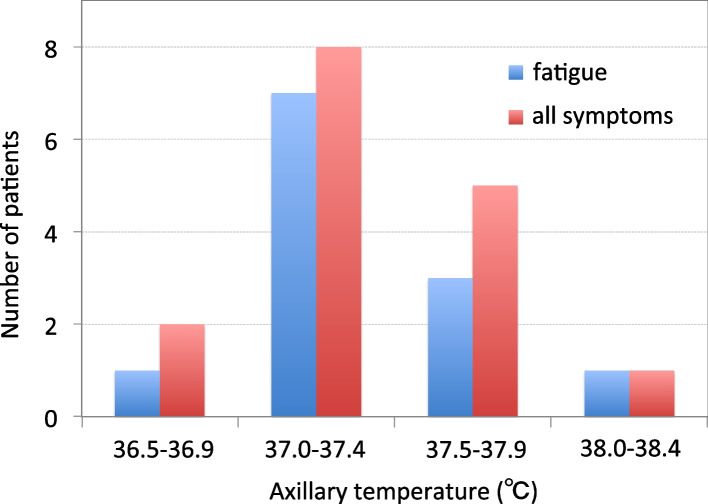
Axillary temperature ranges within which patients with FH experienced exacerbated fatigue (blue column) and all symptoms (red column)

Axillary temperatures at which these symptoms developed, threshold temperatures, ranged from 36.9 °C to 38.0 °C (37.3 ± 0.3 °C on average). In Fig. 1, the axillary temperature ranges at which fatigue and any symptom (shown as all symptoms) developed are shown in increments of 0.5 °C, i.e. 36.5 °C – 36.9 °C, 37.0 °C – 37.4 °C, 37.5 °C – 37.9 °C, and 38.0 °C – 38.4 °C. Both general fatigue and all symptoms were most prevalent at a range of 37.0 °C – 37.4 °C, followed by a range of 37.5 °C – 37.9 °C. Among the 12 patients who experienced general fatigue, it developed at a temperature range of 37.0 °C – 37.4 °C in 7 patients (58.3%) and at 37.5 °C – 37.9 °C in 3 patients (25.0%). Among 16 patients who had any symptoms, those symptoms developed at 37.0 °C – 37.4 °C in 8 patients (50.0%) and at 37.5 °C – 37.9 °C in 5 patients (31.3%).

## Discussion

The results of this study demonstrate that 80% of the patients with FH experienced symptoms related to increased axillary temperature. Among them, 75% reported worsening of general fatigue as a disabling symptom associated with increased axillary temperature. Furthermore, they reported that general fatigue worsened in the temperature range of 37.0 °C to 37.4 °C. They also reported that their fever and these symptoms, except for headache, were not alleviated by NSAIDs. These findings suggest that the reasons patients with FH seek medical treatment when their axillary temperatures are in the 37 °C range are that symptoms such as exacerbation of fatigue occur when the temperature reaches 37 °C and that antipyretic drugs fail to alleviate them.

The mechanisms underlying the reasons patients with FH experience increased fatigue at 37 °C are not fully understood. Several studies have reported average axillary temperature of healthy Japanese samples of 36.9 ± 0.3 °C [4], 36.7 ± 0.3 °C [5], and 36.8 ± 0.3 °C [6]. Therefore, a temperature just above 37.0 °C is within (mean ± 2 SD) or just above the normal range of axillary temperature. Why do patients with FH feel strong fatigue in this temperature range (37.0 °C – 37.4 °C) and feel that it is disabling?

In a previous case, I observed a relationship between axillary temperature and the severity of fatigue in a patient with FH who persistently sought treatment for an axillary temperature in the 37 °C range. Before treatment, she reported that her fatigue level increased remarkably, from 4 to 9 on a numerical rating scale (NRS) of 0 = none to10 = maximum as her axillary temperature increased from 36.8 °C to 37.3 °C. However, one week after receiving tandospirone (30 mg), a 5-hydroxytryptamine (5-HT)1A receptor agonist, she reported a fatigue level of 2 when her axillary temperature was 37.3 °C. Thereafter, the dose of tandospirone was increased to 60 mg, and two weeks after the start of treatment her axillary temperature decreased to less than 37.0 °C. At this time, she reported that she became less concerned about her low-grade fever because she did not feel fatigue at axillary temperatures around 37.0 °C [1, 2]. Based on this finding, it is possible that hypofunctioning of 5-HT in the central nervous system is involved in hyperthermia-associated fatigue and FH itself. However, further studies are necessary to support this hypothesis.

In the present study, 13 (65%) patients visited psychiatrists, and 9 (45%) of them were diagnosed with psychiatric disorders, including depressive and anxiety disorders. This high incidence of comorbid psychiatric disorders is not surprising. Oka K et al. also reported that about half of the patients with FH who visited the outpatient clinic for fever of unknown origin had a psychiatric history, which was significantly more common than febrile patients with organic diseases [7]. Therefore, another possible explanation would be that comorbid psychiatric disorders cause patients to experience increased body temperature and other more disabling physical symptoms [8, 9]. Even if that is not the case, patients with these comorbid psychiatric disorders [10, 11] and physical diseases, such as orthostatic dysregulation [12, 13] and sleep apnea syndrome [14], have been reported to exhibit hyperthermia.

Notably, 13 of our 20 patients (65%) were female. As a woman's basal body temperature changes with the menstrual cycle, it would also be interesting to know whether the menstrual cycle affects their disabling symptoms associated with a high axillary temperature. According to the patient records, no female patient mentioned any relation between her symptoms and her menstrual cycle. However, because they were not specifically asked about that point, it would be premature to conclude that the menstrual cycle does not influence the symptoms associated with the hyperthermia of female patients; thus, further studies are necessary to address this issuse.

There are several limitations in this study. First, the number of subjects is relatively small. The conclusion must be confirmed in a future study with a larger number of subjects. Second, this study evaluated only patients who were referred to my department for treatment. Some patients may not care about a slight increase in axillary temperature. Such patients would not visit a health care facility for treatment, and would not have been included in this study. However, despite these limitations, to the best of my knowledge, this study is the first to demonstrate that patients with FH have symptoms, especially fatigue, when their axillary temperature exceeds 37 °C. This may be the reason patients with FH seek treatment, even though this value is within or just above the normal range of axillary temperature for healthy people.

## Conclusions

In this study, 80% of patients with FH experienced symptoms associated with an increase in axillary temperature. Among the symptoms, worsening of general fatigue was the most common, and it developed in the temperature range of 37.0 °C to 37.4 °C. These findings suggest that the reasons patients with FH with an axillary temperature of 37 °C worry about their temperature and consult their physician to request treatment are that physical symptoms, such as general fatigue, worsen at 37 °C and that most symptoms are not alleviated by antipyretic drugs.

## Data Availability

Data sharing is not applicable.

## Abbreviations

FH: Functional hyperthermia
5-HT: 5-Hydroxytryptamine
NRS: Numerical rating scale
NSAIDs: non-steroidal anti-inflammatory drugs
SD: Standard deviation
